# Influence of Phosphorus Slag on Physical and Mechanical Properties of Cement Mortars

**DOI:** 10.3390/ma13102390

**Published:** 2020-05-22

**Authors:** Min Pang, Zhenping Sun, Ming Chen, Jianlei Lang, Jiayan Dong, Xu Tian, Jiliang Sun

**Affiliations:** 1 School of Materials Science and Engineering, Tongji University, Shanghai 201804, China; pangmin@tongji.edu.cn (M.P.); chenming_tj@126.com (M.C.); 2 Key Laboratory of Advanced Civil Engineering Materials of Ministry of Education, Tongji University, Shanghai 201804, China; 3 Department of Product Design, Zhejiang Da Dongwu Group Construction Co., Ltd., Huzhou 313000, China; langjl_project@126.com; 4 Research and Development Center, Baosen (Shanghai) Environmental Engineering Co., Ltd., Shanghai 200439, China; david_dongjy@163.com (J.D.); tianxu_engineer@126.com (X.T.); sunjiliang_work@126.com (J.S.)

**Keywords:** phosphorus slag, cement mortar, compressive strength, microstructure, carbonation

## Abstract

Influences of phosphorus slag from 10% to 50% (by mass) on the setting time and the water requirement of the normal consistency of cement pastes, flowability, resistance to carbonation, and the compressive strength of cement mortars were investigated. The physical activation by improving fineness and the chemical activation by adding the chemical activator were evaluated by the compressive strength of cement mortars with 30% by mass of phosphorus slag. Hydration heat, X-ray diffraction, and scanning electron microscopy were used to study the microstructure of cement pastes and mortars with 30% by mass of phosphorus slag and the chemical activator. Results showed that the setting time of cement pastes was delayed by phosphorus slag from 10% to 50%. Phosphorus slag had nearly no effects on the water requirement of the normal consistency of cement pastes and the flowability of cement mortars. The resistance to carbonation of cement mortars was decreased by phosphorus slag from 10% to 50% according to the acceleration carbonation. The compressive strength of cement mortars was also decreased by phosphorus slag from 10% to 50% and the low activity of phosphorus slag was concluded based on compressive strength of cement mortars. The effect of the chemical activator on the compressive strength of cement mortars with 30% by mass of phosphorus slag was better than improving fineness of phosphorus slag from 300 m^2^/kg to 450 m^2^/kg. Both hydration heat and cement hydrates were inhibited by phosphorus slag and could be partly compensated by the chemical activator. Loose morphology and propagations of microcracks were found in cement pastes and mortars with 30% by mass of phosphorus slag.

## 1. Introduction

China is the second largest phosphorus reserve-rich country and has become one of the most intensively engaged in phosphorus-processing, which has eventually increased the risk to ecological deterioration from air storage of phosphogypsum and phosphorus slag [1]. Phosphogypsum has been made into belite–ferroaluminate cement products [2], non-autoclaved aerated concrete [3], backfilling materials [4,5], gypsum products [6], combined additives in cement [7]. Phosphorus slag can also be made into backfilling materials [8], porcelain [9], glass-ceramics [10], spherical-CaCO_3_ in the chemical industry [11], cast stone as decorative materials [12], mineral fillers in asphalt binders [13,14], and solidification additives for lead-contaminated soil [15]. However, it is a win-win solution for phosphorus slag to be regarded as the latent SCM (supplementary cementitious material), and thus avoiding environmental pollutions from air storage and CO_2_ emissions from cement usage. Phosphorus slag is one by-product discharged during the extracting process of phosphorus under high temperature electric furnace. Major components in phosphorus slag are SiO_2_ and CaO, minor components are Al_2_O_3_, Fe_2_O_3_, MgO, and P_2_O_5_. Contents of the glass network in granulated phosphorus slag may arrive at 85%~90% because of the high viscosity of the molten slag, similar to granulated blast furnace slag (GGBS) [16].

Under inter-grinding with cement clinkers to make phosphorus slag cement (PSC) it is reported that the slow setting and the low early-strength of PSC cannot be avoided by itself unless using chemical agents [17,18], or blending PSC with GGBS [19] and steel slag [20]. If phosphorus slag could be made into cement clinkers in the unilateral way or the multilateral way between fluorite and steel slag, it would be a good mineralizing agent for nucleation and growth of tricalcium silicate (C_3_S) [21,22]. Directly blended with cement pastes, the setting delay still exists and the hydration heat of cement pastes with 35% by mass of phosphorus slag has been cut down by 49.11% [23]. It is suggested that cement pastes with 40% by mass of phosphorus slag can meet the standard for compressive strength of Type P.O.42.5 cement on the condition that the fineness of cement is increased to 460 m^2^/kg [24]. The early-strength of blended pastes can also be modified if the particle size distribution of phosphorus slag is properly adjusted [25]. The steam-curing condition has been believed to be capable to repair retardations of phosphorus slag [26]. Mechanisms of phosphorus slag on the porosity of blended pastes are studied by transformations of cement hydrates [27] and the fractal theory [28]. Effects of phosphorus slag on mechanical strength and chemical shrinkage are briefly discussed by models along with comparisons to cement pastes with GGBS [29,30].

Many researchers have tried to enhance phosphorus slag by the alkaline activation. The modulus of water glass has great influences on early hydration and compressive strength of alkali-activated phosphorus slag cement [31]. The blended activator of water glass and NaOH is also recommended to be used for phosphorus slag [32]. Rheological behaviors of activated phosphorus slag are strengthened by another blended activator of Ca(OH)_2_ and Na_2_SO_4_ [33]. Attention is put on the efflorescence in alkali-activated phosphorus slag cement [34]. Compressive strength of alkali-activated phosphorus slag cement has been predicted by a temperature–age model [35] and a statistical model [36], tested under different curing conditions [37]. Resistance to freeze–thaw cycles, frost salt attack, and sulfate attack of activated phosphorus slag pastes and mortars are presented by [38,39,40]. Transformations of calcium arsenate waste under solidification by alkali-activated phosphorus slag are studied by [41].

Although the setting delay has been detected in blended concretes, the reduction on hydration heat and resistance to shrinkage of phosphorus slag are advantaged for massive concretes like dams and T-shaped beams of bridges [42,43,44]. Compared to ordinary fineness of 300 m^2^/kg and 391 m^2^/kg in [42,44], effects of superfine phosphorus slag on concretes are tried as well. Modifications on porosity are found by phosphorus slag from 600 m^2^/kg to 800 m^2^/kg [45]. Improvements on resistance to carbonation, chloride penetration, sulfate attack, compressive strength, and splitting tensile strength of concretes are provided by phosphorus slag of 657 m^2^/kg [46]. The cushion effect of phosphorus slag on ASR (alkali–silica reaction) in sleeper-concrete is confirmed according to ASTM C 1260-94 (Mortar-Bar Method) [47]. Resistance to water permeability of concretes can be modified by phosphorus slag [48]. Tensile strength of self-compacting concrete with phosphorus slag is better than plain self-compacting concrete, but lower than self-compacting concrete with fly ash microbeads [49]. Phosphorus slag is effective to improve the workability of concretes compared to fly ash [50].

There are a few research focusing on synergistic effects on concretes with phosphorus slag and other additives. Compressive strength, resistance to frost attack, and chloride penetration of AAPFC concretes (alkali-activated phosphorus slag fly ash cement) are better than plain concretes, but the resistance to carbonation is lower [51]. The initial research of waste clay brick powder and phosphorus slag to produce geopolymer mortars has been started by [52]. The diameter of the most probable pore in reactive powder concrete with phosphorus slag and silica fume is less than 10 nm, which leads to superior mechanical properties and durability [53]. To repair spillways and discharge holes of dams, one type of underwater concretes is developed, which contains phosphorus slag, graphite tailings, fly ash, GGBS, and cellulose ethers [54]. Wear-resisting strength of concretes can be enhanced by fly ash with phosphorus slag [55]. Porosity of cement pastes with phosphorus slag and ferronickel slag is inferior to cement pastes with fly ash at early ages [56]. One type of road lining materials is invented by [57], which is made up of phosphorus slag, lime, and fly ash.

Nowadays, products of polymer cement concrete (PCC) are widely applied and some types of PCC can be classified into dry mortars [58]. Basic additives in dry mortars are cellulose ethers and re-dispersible polymer powders which are responsible for water retention of fresh mortars and adhesive strength of hardened mortars [59,60]. As one type of dry mortars, self-leveling mortars can be specially designed for phosphorus slag. Unfortunately, there are almost no studies about the properties of cement mortars with phosphorus slag, not to mention influences of curing conditions and bleeding during the fresh state [61,62]. Therefore, it is necessary to find out.

In this study, a basic research was made to evaluate effects of phosphorus slag from 10% to 50% (by mass) on behaviors of cement mortars. The setting time and water requirement of normal consistency for cement pastes with phosphorus slag were studied as well as the flowability of fresh mortars. The resistance to carbonation of cement mortars with phosphorus slag was tested by the acceleration carbonation. The compressive strength of cement mortars with phosphorus slag was investigated and the pozzolanic activity of phosphorus slag was assessed by one traditional system based on results of compressive strength. To activate phosphorus slag, the physical activation by increasing fineness and the chemical activation by adding the chemical activator were attempted. Mineralogy and hydration heat of cement pastes with phosphorus slag and the chemical activator were presented by X-ray diffraction (XRD) and an isothermal calorimeter. Morphology of cement pastes and mortars with phosphorus slag and the chemical activator were observed by scanning electron microscopy (SEM).

## 2. Materials and Methods

### 2.1. Materials and Mix Proportions

**Figure 1:**
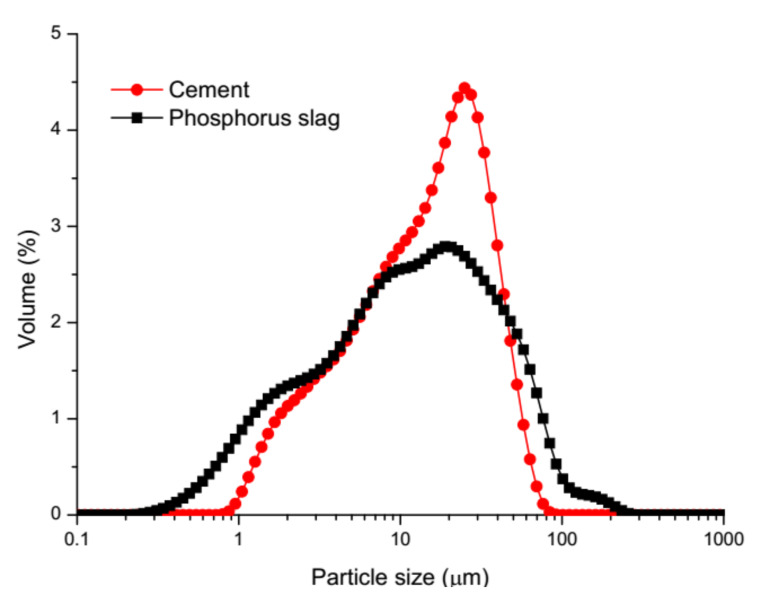
Particle size distributions of cement and phosphorus slag.

**Figure 2:**
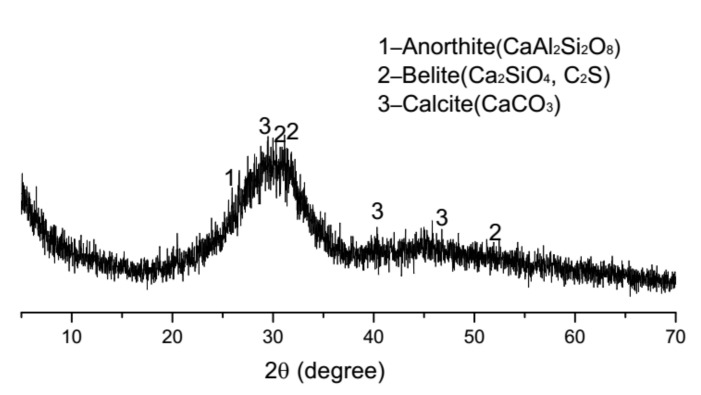
XRD pattern of phosphorus slag.

The P.O.42.5 cement (Conch Cement Corp., Anhui, China) and phosphorus slag (Hubei, China) were used. The fineness of P.O.42.5 cement and grinded phosphorus slag were 336 m^2^/kg and 300 m^2^/kg, respectively. Their particle size distributions are shown in Figure 1. The density of phosphorus slag and P.O.42.5 cement were 2856 kg/m^3^ and 3100 kg/m^3^, respectively. The chemical compositions of P.O.42.5 cement and grinded phosphorus slag are listed in Table 1. The XRD pattern of phosphorus slag is shown in Figure 2. Ordinary river sand was used for aggregates, with a fineness modulus of 2.4 and a bulk density of 1450 kg/m^3^. Tap water was used for mixing the samples. The mix proportions of the mortar samples are listed in Table 2. Curing conditions of samples were at 20 ± 1 °C and (90 ± 1%) RH.

### 2.2. Performance of Cement Pastes and Fresh Mortars

The setting time and water requirement of normal consistency of cement pastes with phosphorus slag were evaluated according to GB/T 1346-2011. Mix proportions of paste samples are listed in Table 3. It was noteworthy that there were two methods to test the water requirement of the normal consistency for cement pastes in GB/T 1346-2011. The method in this experiment was to adjust the water consumption. The fluidity of fresh mortars with phosphorus slag was tested according to GB/T 2419-2005, which was called the flow table test. Details of the flow table test were as follows. Fresh mortars were cast into a trapeziform metal container above a rounded table, and then vibrated 25 times for 25 s, moving freely without the container until plates of fresh mortars stopped. The diameters of fresh mortar plates were recorded, and the average value was determined for fluidity of fresh mortars.

### 2.3. Compressive Strength

The compressive strength of mortars was measured according to GB/T 17671-1999 at 3 days, 28 days, and 90 days. Each result was the average value of 5 specimens (40 mm × 40 mm × 160 mm). Tests were undertaken at a loading rate of (2400 ± 200) N/s. One traditional evaluation system of pozzolanic activity was cited to phosphorus slag. This evaluation system was officially proposed by Professor Xincheng Pu for refereeing contributions of SCMs to compressive strength of high strength concrete and high performance concrete [63]. In this evaluation system, the pozzolanic activity of certain SCM was defined by Equation (1) to Equation (5). In Equation (1), F_RC_ is the relative compressive strength, F_C_ is the original compressive strength, and Q is the percentage of cement dosage. In Equation (2), F_PC_ is the compressive strength of the pozzolanic activity and F_BC_ is the compressive strength of plain sample. In Equation (3), K is the coefficient on the compressive strength of the pozzolanic activity. In Equation (4), P_P_ is the contribution of the pozzolanic activity to the compressive strength. In Equation (5), P_H_ is the contribution of the cement hydration to the compressive strength.

(1) $$F_{\mathrm{RC}}=\frac{F_{C}}{Q}$$

(2) $$F_{\mathrm{PC}}=F_{\mathrm{RC}}-F_{\mathrm{BC}}$$

(3) $$K=F_{\mathrm{RC}}/F_{\mathrm{BC}}$$

(4) $$P_{P}=F_{\mathrm{PC}}/F_{\mathrm{BC}}$$

(5) $$P_{H}=F_{\mathrm{BC}}\;/F_{\mathrm{PC}}$$

### 2.4. Resistance to Carbonation

Procedures of carbonation tests were performed to GBT50082-2009 (the acceleration carbonation). Samples were cured for 26 days and then dried for 48 h in an oven at 60 °C. Samples were entirely coved by paraffin, only opening two vertical interfaces of the square section. Treated samples were placed in the carbonation chamber. The condition of the carbonation chamber was firmly kept with a CO_2_ concentration of 20 ± 3%, temperature of (20 ± 2) °C, and relative humidity of 70% ± 5%. Samples were taken out after carbonation of 1 day, 3 days, and 7 days, broken vertically along opening interfaces, and sprayed by phenolphthalein solutions (0.1 mol/L) for carbonation depth.

### 2.5. Activation of Phosphorus Slag

The physical activation and the chemical activation were executed. In the physical activation, the fineness of phosphorus slag was grinded from 300 m^2^/kg to 350 m^2^/kg, 400 m^2^/kg, and 450 m^2^/kg. Effects of the physical activation were checked by compressive strength of cement mortars with 30% by mass of phosphorus slag at early ages (3 days, 7 days, 28 days). In the chemical activation, one activator was used which contained Al_2_(SO_4_)_3_, Na_2_SO_4_, CaCl_2_, Ca(OH)_2_ as 1:1:1:1 by mass. Effects were also checked by compressive strength of cement mortars with 30% by mass of phosphorus slag (300 m^2^/kg) and 1% activator (by mass to phosphorus slag).

### 2.6. Microstructure Analysis

Cement pastes with 30% by mass of phosphorus slag, plain pastes, and cement pastes with 30% by mass of phosphorus slag and the chemical activator were produced at w/c = 0.5. Under the same conditions, these pastes were cured for 3 days and 28 days (20 mm × 20 mm × 20 mm). The mineralogical phases were determined by XRD analysis. XRD analysis was performed by the X-ray diffraction equipment (Bruker, Karlsruhe, Germany) of Rigaku-D/max2550VB3+, from 5° to 75° at 5°/min with a Cu Kα radiation. XRD peaks were automatically calculated by the software of MDI Jade 6.5. (version 6.5, MDI, Livermore, CA, USA) SEM analysis was tested by the equipment of FEI Quanta 200 FEG (FEI, Hillsboro, OR, USA). The evolution of hydration heat was measured by the isothermal calorimeter (TAM AIR C80, Thermometric, Järfälla, Sweden).

## 3. Results and Discussion

### 3.1. Behavior of Cement Pastes and Fresh Mortars

**Figure 3:**
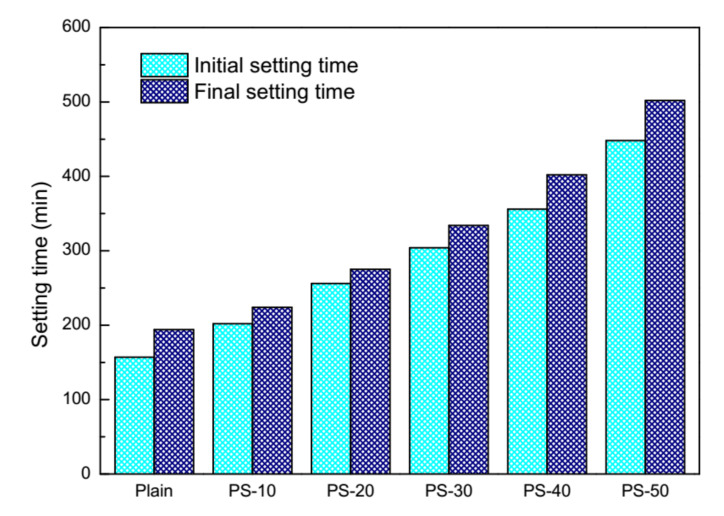
Effects of phosphorus slag on the setting time.

Effects of phosphorus slag on the setting time of cement pastes are demonstrated in Figure 3. It can be seen that both the initial setting time and the final setting time of cement pastes are delayed by phosphorus slag. The initial setting time of plain pastes starts at 157 min, arriving at 202 min, 256 min, 304 min, 356 min, and 448 min along with phosphorus slag from 10% to 50% (by mass). The final setting time of plain pastes is accordingly prolonged from the beginning of 194 min to 224 min, 275 min, 334 min, 402 min, and 502 min. The gap between the initial setting time and the final setting time of plain pastes is 37 min, those of blended pastes are 22 min, 19 min, 30 min, 46 min, and 54 min, respectively. The correlation between the setting time of fresh pastes and the dosage of phosphorus slag can be regarded as the linear growth, which is similar to phosphorus slag cement [18] and phosphorus slag concrete [42].

**Figure 4:**
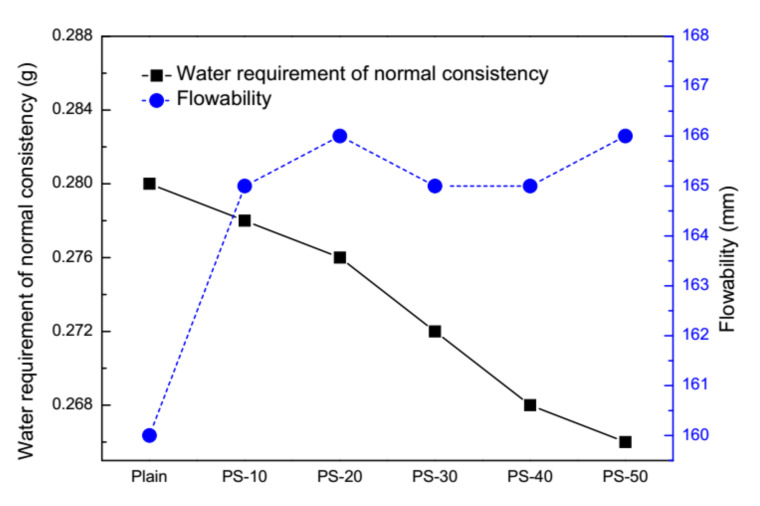
Effects of phosphorus slag on water requirement of normal consistency and flowability.

The flowability of fresh mortars and water requirement of normal consistency for cement pastes is illustrated in Figure 4. The flowability of fresh mortars is slightly increased by phosphorus slag, but different dosages cannot make remarkable fluctuations. The value of plain pastes is 160 mm, the value of blended pastes with 10% by mass of phosphorus slag is 165 mm, and the rest of the others are 166 mm, 165 mm, 165 mm, and 166 mm.

The water requirement of normal consistency for cement pastes is decreased by phosphorus slag in a marginal manner. As adjustments of water consumption are used here to fix the consistency of cement pastes, this physical parameter means the homogeneity degree of components in fresh pastes and the capacity of resistance to bleeding and stratification to a certain extent. The total decrease of water requirement is only 0.014 g, from 0.28 g of plain pastes to 0.266 g of cement pastes with 50% by mass of phosphorus slag. As the low activity of phosphorus slag is mentioned by [18,23,24], it can be assumed that in this situation the low activity of phosphorus slag cannot consume large quantities of water to have a pozzolanic reaction at early ages, and thus leaving the water requirement nearly unchanged.

### 3.2. Compressive Strength

**Figure 5:**
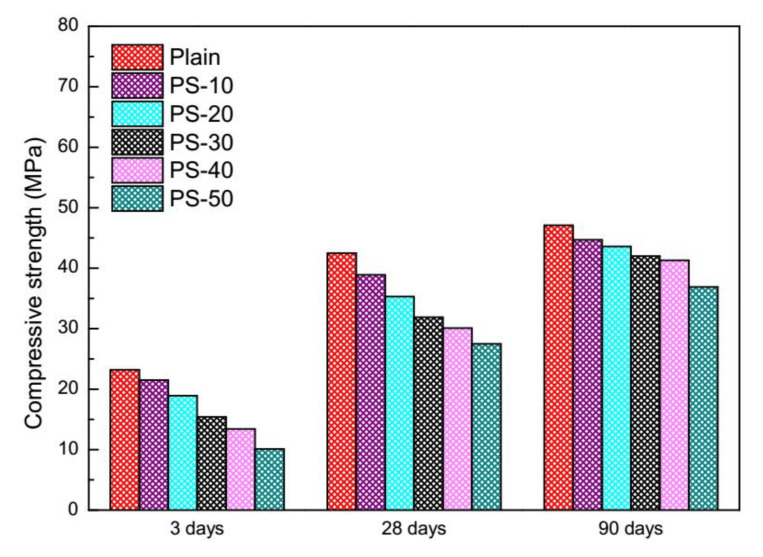
Compressive strength of cement mortars with phosphorus slag at different ages.

Compressive strength of plain mortars and blended mortars at 3 days, 28 days, and 90 days are shown in Figure 5. At an age of three days, the compressive strength of plain mortars is decreased by phosphorus slag and the descending order is regularly followed to phosphorus slag from 10% to 50% (by mass). The weakest compressive strength of blended mortars with 50% by mass of phosphorus slag is 43.5% to that of plain mortars. The broken situation of phosphorus slag on compressive strength is similar at an age of 28 days, as well as an age of 90 days.

At an age of 28 days, the compressive strength of blended mortars with 50% by mass of phosphorus slag is 64.7% to that of plain mortars. At an age of 90 days, the compressive strength of blended mortars with 50% by mass of phosphorus slag rises to 78.3%. Degradations of phosphorus slag on compressive strength of concretes were presented by [42] in which the descending orders at 28 days and 90 days showed the same trends.

Integrate performance of the pozzolanic activity of phosphorus slag at different ages are evaluated in Table 4, Table 5 and Table 6. At an age of three days, the compressive strength of the pozzolanic activity (F_PC_) and contribution of the pozzolanic activity to compressive strength (P_P_) straightly decreases to negative values along with phosphorus slag from 10% to 50% (by mass). This trend reveals that phosphorus slag nearly makes no contributions to the compressive strength of mortars, or even postpones the development of compressive strength. Therefore, the coefficient on compressive strength of the pozzolanic activity (K) shows a downside trend. At an age of 28 days, all of F_PC_, P_P_, and K have increased, which means the pozzolanic activity of phosphorus slag starts to make efforts. At an age of 90 days, all of F_PC_, P_P_, and K imply that the pozzolanic activity of phosphorus slag continues to be helpful.

According to Equations (1) and (5), the values of F_RC_ and the values of P_H_ can be used as an indicator to the hydration degree of blended pastes with phosphorus slag. Among all three tables, all of F_RC_ in Table 6 are the highest, which means the deeper hydration degree of blended mortars appears at 90 days. A few irregular trends of F_RC_ and P_H_ in Table 4, Table 5 and Table 6 should be noted. These situations are explained in the original text [63]. This pozzolanic evaluation system may not be fully suitable for some types of SCMs because of the physical effects that are not separated.

### 3.3. Carbonation Depth

**Figure 6:**
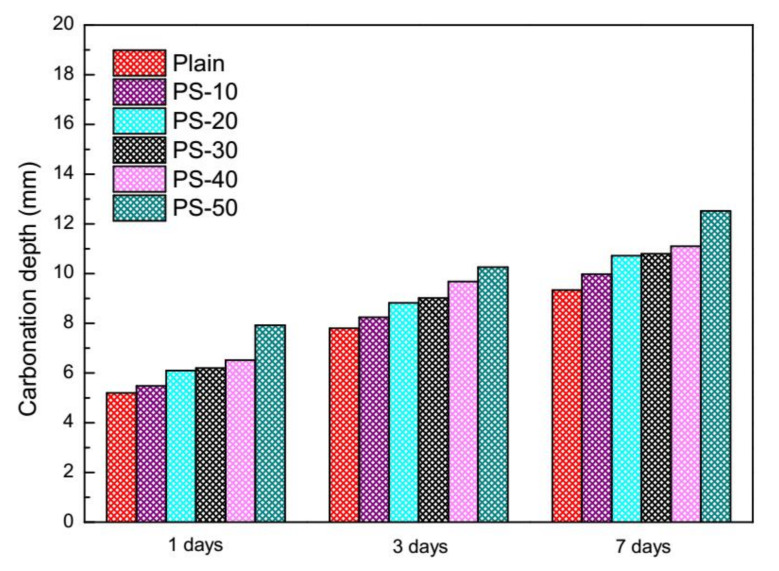
Carbonation depth of cement mortars with phosphorus slag at different exposure times.

Carbonation depth of plain mortars and blended mortars are illustrated in Figure 6. One can see that the carbonation depth of blended mortars during the exposure times are deeper than those of plain mortars, which implies that resistance to carbonation of plain mortars is reduced by phosphorus slag. The ascending order of carbonation depth of blended mortars is regular to phosphorus slag from 10% to 50% (by mass). Damages on resistance to carbonation of concretes at early ages of three days and seven days were found by [50], but beneficial effects can be gained at later ages on the condition that the fineness of phosphorus slag are improved.

Due to the changes caused by carbonation in phase assemblage [64], porosity and pore solution [64,65,66], characteristics of carbonation zone and front [67], effects of water to cement ratio [66], attentions and restrictions of SCMs [68,69,70,71,72], and inferior resistance to carbonation of blended mortars may come from three aspects: (1) the setting delay and the low activity of phosphorus slag result in a loose morphology in mortars for CO_2_ to easily penetrate; (2) the experimental conditions of the high water to cement ratio of 0.5 and the acceleration carbonation are harmful; (3) the high volume of phosphorus slag ranges from 30% to 50% (by mass).

### 3.4. Physical and Chemical Activation

**Figure 7:**
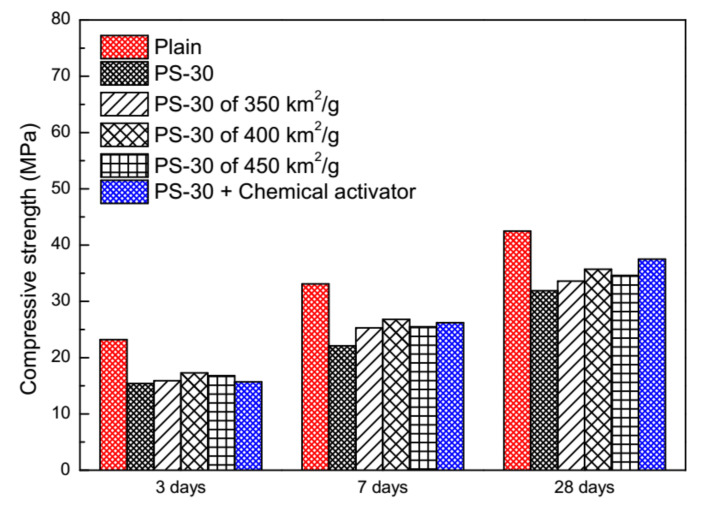
Compressive strength of cement mortars with 30% by mass of phosphorus slag under the physical activation and the chemical activation.

The physical activation and the chemical activation are evaluated by the compressive strength of blended mortars with 30% by mass of phosphorus slag at early ages (Figure 7). After the fineness activated, the compressive strength of blended mortars with phosphorus slag of 400 m^2^/kg is better than the rest of others at 3 days, 7 days, and 28 days. However, they are only 74.6%, 81.2%, and 84.8% to those of plain mortars. Although lower than the compressive strength of plain mortars either, the chemical activator shows its effects on the compressive strength of blended mortars with phosphorus slag of 300 m^2^/kg, 67.8% to that of plain mortars at an age of three days and 88.2% at last. The lowest compressive strength of blended mortars is the one with phosphorus slag of the coarsest fineness (300 m^2^/kg), which is 66.4%, 66.7%, and 75.1% to those of plain mortars at different ages.

Both the physical activation and the chemical activation are effective, but the chemical activation is better. This situation is in accordance with [17,18] in which several types of fast-setting/early-strength chemical agents were used to compensate the low strength of PSC at early ages. The activity of the glass network in SCMs can be more easily released by the chemical corrosion than the physical grinding, just as granulated blast furnace slag [73,74,75]. A better physical activation can be obtained by improving fineness of cement and phosphorus slag together [24].

### 3.5. Microstructure Analysis

**Figure 8:**
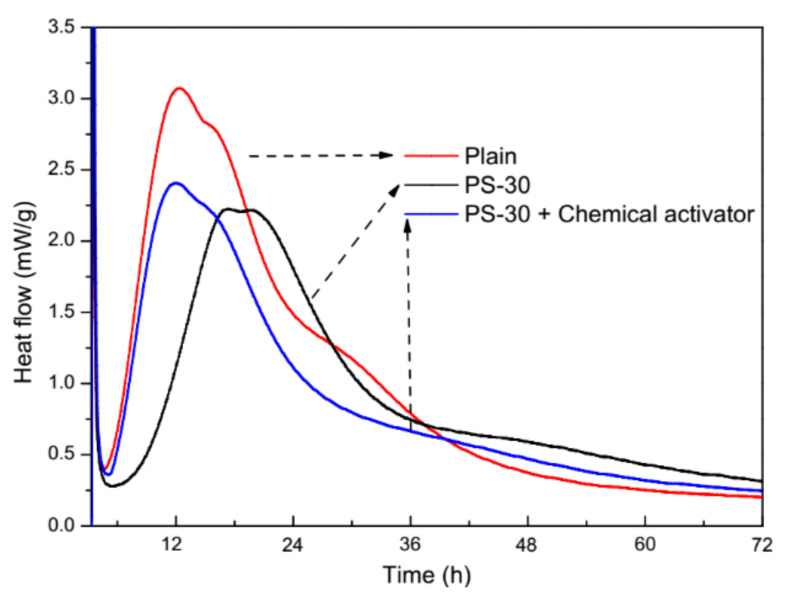
Hydration heat flow curves of plain pastes and cement pastes with 30% by mass of phosphorus slag and the chemical activator.

Hydration heat flow curves of plain pastes and blended pastes are demonstrated in Figure 8. The peak in the hydration heat flow curve of plain pastes is higher and shorter in time than the peak in the curve of blended pastes with 30% by mass of phosphorus slag, which attributes to the setting delay of phosphorus slag. After the chemical activator is added, the peak in the hydration heat flow curve is elevated higher and shorter in time, nearly the same to that of plain pastes. Changes in the hydration heat flow curves in Figure 8 have revealed that the chemical activator can compensate the setting delay of phosphorus slag on cement pastes.

**Figure 9:**
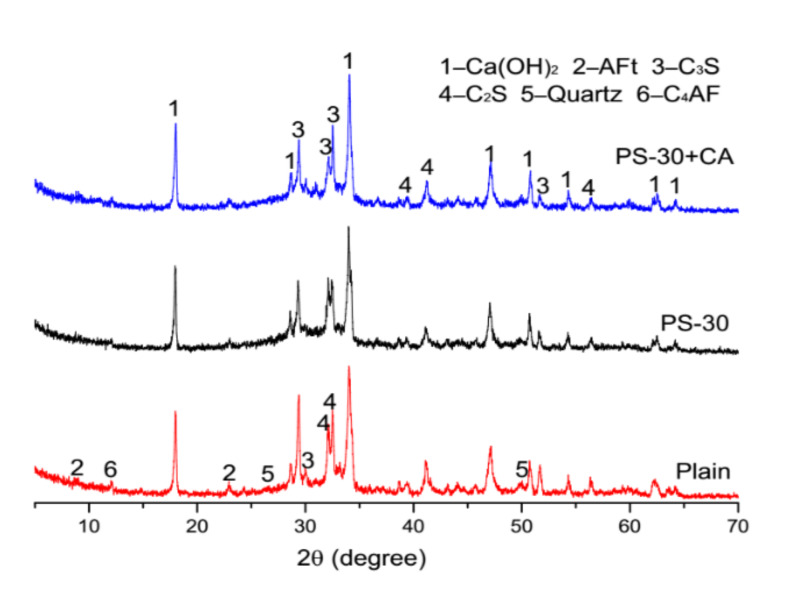
XRD patterns of plain pastes and cement pastes with 30% by mass of phosphorus slag and the chemical activator at 3 days.

**Figure 10:**
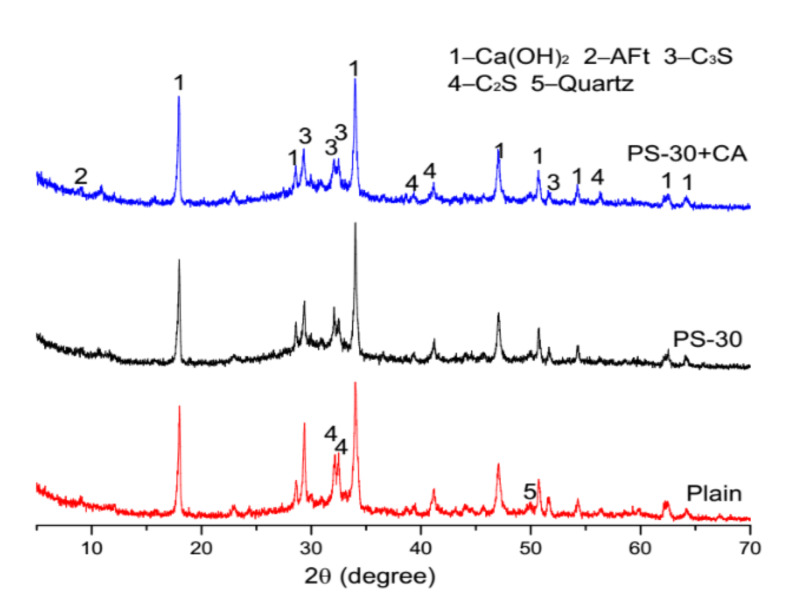
XRD patterns of plain pastes and cement pastes with 30% by mass of phosphorus slag and the chemical activator at 28 days.

XRD patterns of plain pastes and blended pastes at 3 days and 28 days are demonstrated in Figure 9 and Figure 10. At an age of three days, both the intensity of ettringite (AFt) and the intensity of brownmillerite (C_4_AF) are decreased by phosphorus slag, but promoted by the chemical activator. At an age of 28 days, the intensity of ettringite (AFt) is increased by the chemical activator in blended pastes with 30% by mass of phosphorus slag. The considerable declines of the intensity of tricalcium (C_3_S) and the intensity of decalcium (C_2_S) are observed in blended pastes with 30% by mass of phosphorus slag and the chemical activator, which means cement hydrates are more aggressive.

**Figure 11:**
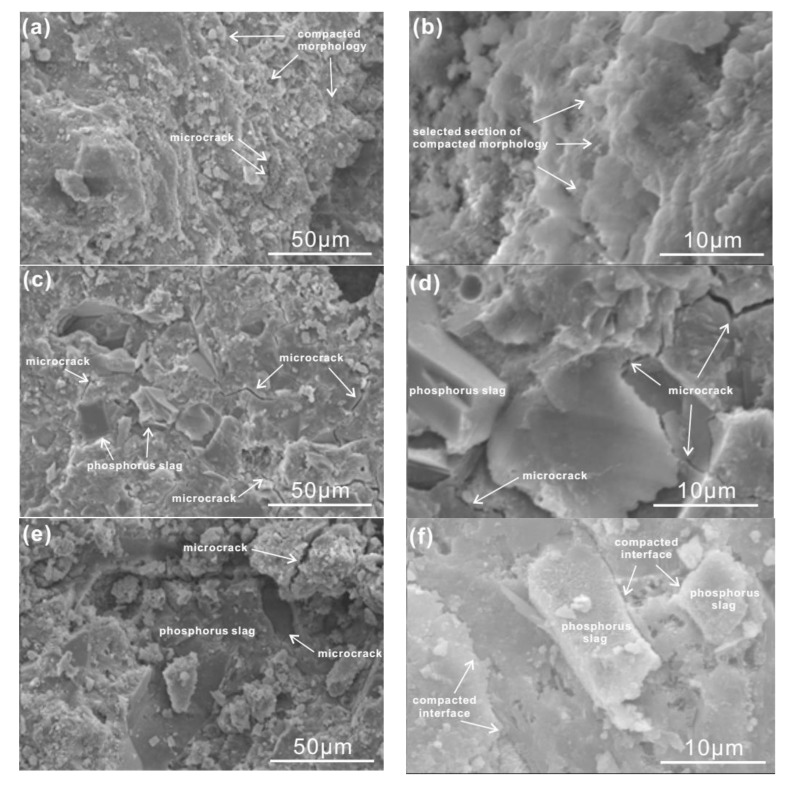
SEM images of cement pastes at different ages. (**a**,**b**) Plain pastes at 3 days and 28 days; (**c**,**d**) cement pastes with 30% by mass of phosphorus slag at 3 days and 28 days; (**e**,**f**) cement pastes with 30% by mass of phosphorus slag and the chemical activator at 3 days and 28 days.

**Figure 12:**
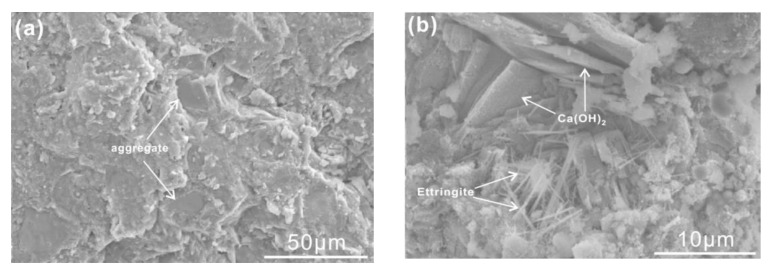

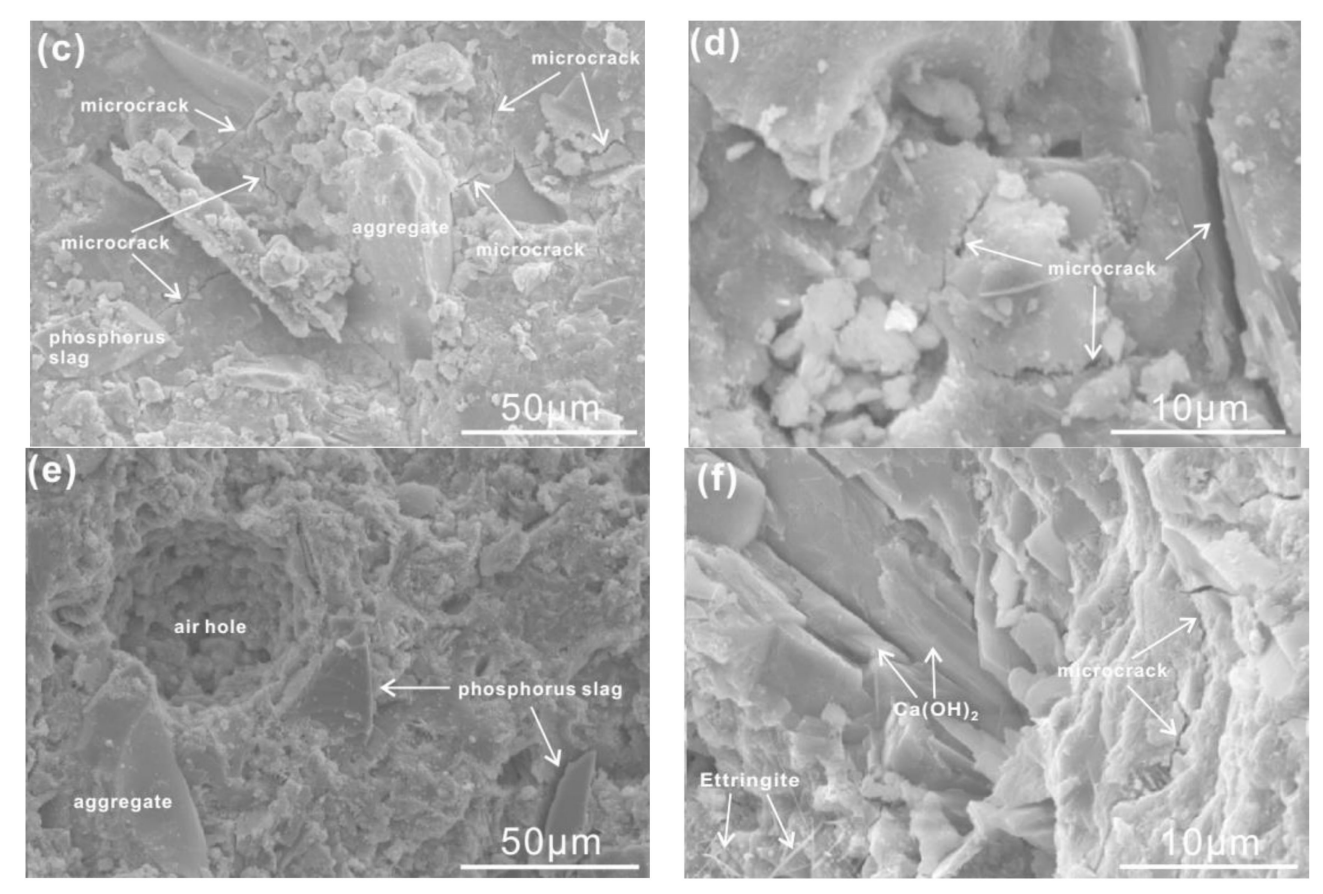
SEM images of cement mortars at different ages. (**a**,**b**) Plain mortars at 3 days and 28 days; (**c**,**d**) cement mortars with 30% by mass of phosphorus slag at 3 days and 28 days; (**e**,**f**) cement mortars with 30% by mass of phosphorus slag and the chemical activator at 3 days and 28 days.

SEM images of blended pastes and mortars at 3 days and 28 days are demonstrated in Figure 11 and Figure 12, respectively. From the morphology of plain pastes at 3 days and 28 days in Figure 11, it is clear that bulk cement hydrates are compacted and there are little microcracks. Microcracks can be obviously seen in Figure 11c,d. The morphological characteristic of blended pastes is the intergrowth between cement hydrates and phosphorus slag, which leads to inferior interfaces popular for microcracks. Consequently, loose morphology and plenty of microcracks result in worse compressive strength and deeper carbonation depth. This situation has been partly repaired by the chemical activator in Figure 11e,f.

From the morphology of plain mortars at 3 days and 28 days in Figure 12, one can see that aggregates are closely embedded in bulk cement hydrates. Stack layers of Ca(OH)_2_ and needle-like ettringite (AFt) have implied that cement hydrates are almost the same as usual. Comparatively, phosphorus slag has provoked microcracks, especially surrounding aggregates (Figure 12c,d). Microcracks are remarkably extinguished in Figure 12e,f. Meanwhile, crystals of Ca(OH)_2_ and ettringite (AFt) in selected sections are the same as those in plain mortars, owing to an accelerated hydration by the chemical activator.

## 4. Conclusions

This article mainly aimed at effects of phosphorus slag from 10% to 50% (by mass) on the setting time and water requirement of normal consistency for cement pastes, flowability, resistance to carbonation, and compressive strength of cement mortars, as well as the physical activation and the chemical activation on cement mortars with 30% by mass of phosphorus slag at early ages. Cement pastes and mortars with 30% by mass of phosphorus slag and the chemical activator were revealed by hydration heat, X-ray diffraction (XRD), and scanning electron microscopy (SEM).

The setting time of cement pastes was delayed by phosphorus slag and the delay order was followed to the increase of phosphorus slag from 10% to 50% (by mass). The time interval between the initial setting time and the final setting time was prolonged according to the increase of phosphorus slag from 10% to 50% (by mass). The water requirement of normal consistency for cement pastes and the flowability of cement mortars were not fluctuated by the increase of phosphorus slag from 10% to 50% (by mass).

The resistance to carbonation of cement mortars was declined by the increase of phosphorus slag from 10% to 50% (by mass) according to the acceleration carbonation. The compressive strength of cement mortars was decreased by the increase of phosphorus slag from 10% to 50% (by mass), especially at an early age of three days. The low activity of phosphorus slag was concluded based on compressive strength of cement mortars.

The physical activation by improving fineness of phosphorus slag from 300 m^2^/kg to 450 m^2^/kg was less effective on the compressive strength of cement mortars with 30% by mass of phosphorus slag than the chemical activation by adding the chemical activator.

Although phosphorus slag could inhibit the hydration process of cement pastes according to hydration heat flow curves from 0 to 72 h and XRD patterns at 3 days and 28 days, and provoke microcracks to propagate according to SEM images, compensations could be made by adding the chemical activator which is a potential method for this by-product in cement-based materials.

## Figures and Tables

**Table 1:** Chemical compositions of P.O.42.5 cement and phosphorus slag (wt %).

Binders	SiO_2_	CaO	Al_2_O_3_	Fe_2_O_3_	MgO	Na_2_O	K_2_O	TiO_2_	SO_3_	P_2_O_5_	Loss on Ignition
Cement	20.8	61.3	6.34	3.07	1.03	0.21	0.85	0.29	2.29	−	2.01
PS	40.8	45.7	2.57	0.41	3.32	0.38	1.01	0.22	1.56	3.91	0.52

**Table 2:** Mix proportion of mortar samples (kg/m^3^).

Sample	Cement	Phosphorus Slag	Sand	Water	Chemical Activator
Plain	500	0	1500	250	0
PS-10	450	50	1500	250	0
PS-20	400	100	1500	250	0
PS-30	350	150	1500	250	0
PS-40	300	200	1500	250	0
PS-50	250	250	1500	250	0
PS-30 of 350 km^2^/g	350	150	1500	250	0
PS-30 of 400 km^2^/g	350	150	1500	250	0
PS-30 of 450 km^2^/g	350	150	1500	250	0
PS-30 + Chemical activator	350	150	1500	250	1.5

**Table 3:** Mix proportion of paste samples (g).

Sample	Cement	Phosphorus Slag	Water
Plain	500	0	140
PS-10	450	50	139
PS-20	400	100	138
PS-30	350	150	136
PS-40	300	200	134
PS-50	250	250	134

**Table 4:** Integrate performance of the pozzolanic activity of phosphorus slag in cement mortars at 3 days.

Sample	F_RC_ (MPa)	F_PC_ (MPa)	K	P_P_	P_H_
Plain	0.232	0.000	1.000	0.0	100.0
PS-10	0.239	0.007	1.030	2.9	97.1
PS-20	0.236	0.004	1.018	1.8	98.2
PS-30	0.220	−0.012	0.948	−5.5	105.5
PS-40	0.223	−0.009	0.963	−3.9	103.9
PS-50	0.202	−0.030	0.871	−14.9	114.9

**Table 5:** Integrate performance of the pozzolanic activity of phosphorus slag in cement mortars at 28 days.

Sample	F_RC_(MPa)	F_PC_(MPa)	K	P_P_	P_H_
Plain	0.425	0.000	1.000	0.0	100.0
PS-10	0.432	0.008	0.957	1.7	104.5
PS-20	0.441	0.016	1.038	3.7	96.3
PS-30	0.456	0.031	1.072	6.7	93.3
PS-40	0.502	0.077	1.180	15.3	84.7
PS-50	0.550	0.125	1.294	22.7	77.3

**Table 6:** Integrate performance of the pozzolanic activity of phosphorus slag in cement mortars at 90 days.

Sample	F_RC_(MPa)	F_PC_(MPa)	K	P_P_	P_H_
Plain	0.471	0.000	1.000	0.0	100.0
PS-10	0.497	0.026	1.054	2.4	97.6
PS-20	0.545	0.074	1.157	6.4	93.6
PS-30	0.600	0.129	1.274	10.1	89.9
PS-40	0.688	0.217	1.461	14.9	85.1
PS-50	0.738	0.267	1.567	17.0	83.0
